# Role of ultrasound in the diagnosis of primary and recurrent dermatofibrosarcoma protuberans

**DOI:** 10.1186/s12885-021-08476-2

**Published:** 2021-08-10

**Authors:** Min-Hong Zou, Qing Huang, Ting Yang, Ye Jiang, Luan-jing Zhang, Yang Xie, Rong-Qin Zheng

**Affiliations:** 1 Department of Ultrasound, The Third Affiliated Hospital, Sun Yat-Sen University, 600 Tianhe Road, Guangzhou, China; 2 Department of General Surgery, Guangzhou Digestive Disease Center, Guangzhou First People’s Hospital, The Second Affiliated Hospital of South China University of Technology, Guangzhou, Guangdong China; 3 Department of Nuclear Medicine, The Third Affiliated Hospital, Sun Yat-Sen University, Guangzhou, China; 4 Department of Pathology, The Third Affiliated Hospital, Sun Yat-Sen University, Guangzhou, China; 5 Department of Dermatology, The Third Affiliated Hospital, Sun Yat-Sen University, Guangzhou, China

**Keywords:** Dermatofibrosarcoma protuberans, Primary, Recurrent, Ultrasound

## Abstract

**Background:**

Dermatofibrosarcoma protuberans (DFSP) is a rare, low- to intermediate-grade sarcoma, which represents a diagnostic imaging challenge.

This study aimed to analyze the clinical and ultrasound features of primary and recurrent DFSP to improve the diagnosis.

**Methods:**

Clinical, imaging, and pathological data from a total of 58 patients (23 patients with primary DFSP and 35 patients with recurrent DFSP) were retrospectively reviewed.

**Results:**

There was no statistically significant difference in age, sex, tumor size, or echogenicity between the two groups. Most of the primary DFSP lesions involved the overlying dermis and hypodermis, while most of the recurrent DFSP lesions were fixated to more deeply seated structures at the original surgical incision. Red nodules on the skin were found more frequently in the primary group. There were statistically significant differences in the type of lesion and ultrasound tumor morphology (*p* < 0.050). The lesions in the primary group showed more tentacle-like projections or a “claw” sign, while the lesions in the recurrent group were more commonly oval, lobulated, and irregularly shaped. Hypervascularity was common in both groups.

**Conclusions:**

For primary DFSP, a slow-growing, red nodule on the skin involving the overlying dermis and hypodermis, more frequently a hypoechoic mass with tentacle-like projections or a “claw” sign, was observed. For recurrent DFSP, palpable subcutaneous nodules or subcutaneous masses at the original surgical incision and oval, lobulated, and irregularly shaped lesions were more commonly observed. This may be useful for improving diagnostic accuracy.

## Introduction

Dermatofibrosarcoma protuberans (DFSP) is a rare, low- to intermediate-grade sarcoma that was first named by Hoffman in 1925. The lesion is derived from dermal fibroblasts but also has a propensity for infiltrating the underlying tissue [1], including fat, muscle, and fascia, which makes it difficult to completely remove the tumor and leads to high recurrence rates after surgery [2].

DFSP represents a diagnostic imaging challenge. Ultrasound (US), computed tomography (CT), and magnetic resonance imaging (MRI) are the most commonly utilized imaging modalities for DFSP diagnosis and assessment. Many reports have described the imaging findings of DFSP based on MRI or CT [3]. MRI/CT is useful for assessment of the extent of involvement, particularly with large and atypical primary lesions or recurrent disease [4]. However, DFSP is typically small and superficial. Diagnostic US examinations, especially high-frequency ultrasonography (HFUS), remain superior in the assessment of superficial soft-tissue masses due to their high-resolution images of superficial tumors [5]. Color Doppler US, which can produce a map of tumoral vessels, is considered one of the most sensitive methods to detect signs of neovascularity in evaluations of nodular skin lesions [6, 7]. Additionally, US examinations are quick, accessible, and inexpensive, and the data can be collected in real time. There have been several reports on the US features of DFSP [8–11]. However, few cases have been included in these studies. Thus, further studies are needed. The differential diagnosis of DFSP and other superficial tumors is very important because the treatment strategies and prognoses differ. Receiving standard tumor resection for the first time is the key to reducing local DFSP recurrence.

The aim of our study was to retrospectively analyze the clinical and US features of primary and recurrent DFSP to identify features that provide diagnostic value and improve diagnostic accuracy.

## Materials and methods

### Patients

This was a retrospective study approved by the Department of Ultrasound of the Third Affiliated Hospital, Sun Yat-Sen University. The complete clinical, imaging and pathological data of 58 patients with DFSP (23 patients with primary disease at our institution and 35 patients with recurrent disease who were referred to us subsequently after incomplete resection in another hospital) were retrospectively studied from 2010 to 2020. Recurrent DFSP excluded residual patients with an incomplete excision. Postoperative ultrasonography was performed every 3 months. Other patients without complete imaging data were excluded. The entire study protocol was approved by our institutional review board. Written informed consent was obtained from all patients or patients’ parents/guardians for use of their clinical data. The inclusion criteria were a final diagnosis of DFSP confirmed by pathology and immunohistochemistry after surgical resection.

### US imaging and image analysis

Because DFSP is a rare tumor, the patient inclusion and data collection periods were long. Multiple real-time high-frequency US systems, including the IU-22 (Philips), Logic E9 (General Electric Company), Aplio 500 (Toshiba Medica System), MyLab Class C (Esaote, Genoa, Italy) and Resona 7S (Mindray), were used.

US images were reviewed by two fellowship-trained doctors with > 5 years of experience in musculoskeletal US who were blinded to the pathological information of all cases. They reviewed the provided images through our picture archiving and communication system (PACS). Discrepancies were resolved by consensus. The following imaging features of each tumor were assessed for all imaging studies: size, location, internal echogenicity, tumor morphology, and vascular information on color Doppler flow imaging (CDFI). Echogenicity of the lesion was compared to that of the adjacent subcutaneous muscle and categorized as hypoechoic, mixed, or hyperechoic. The CDFI diagnostic criteria were as follows: when vascular data showed Adler 0 or 1 [12], we judged the vascularization as not rich, and when the data showed the opposite, we judged the vascularization as rich.

### Histopathology and immunohistochemical examination

All 58 patients underwent surgery. The pathological diagnosis was based on surgical specimens. Paraffin sections (4 μm) were used for hematoxylin and eosin (H&E) staining and immunohistochemical examination (including CD34). Microscopic sections were reviewed by two pathologists with over 16 years of experience in hepatic pathology to confirm the diagnosis.

### Statistical analysis

Statistical analysis was performed using SPSS for Microsoft Windows (version 22.0, SPSS, Chicago, IL, USA). For the between-group comparisons, quantitative data are expressed as the mean ± standard deviation (SD). Categorical data were compared using the Kaplan-Meier method or Fisher’s exact test. Measurement data are presented as the means ± SDs. A *P* value less than< 0.050 was considered statistically significant.

## Results

### Patient clinical features

A total of 58 patients diagnosed with DFSP from 2010 to 2020 were included in this retrospective study. All patients were divided into two groups: the primary DFSP group and the recurrent DFSP group. The clinical characteristics of the patients with DFSP are summarized in Table 1.

**Table 1:** Clinical characteristic of DSFP patients

Characteristic	Primary DSFP (*n* = 23)	Recurrent DSFP (*n* = 35)	*P*-value
Age (years)	42.27 ± 10.86 (25–64)^a^	38.09 ± 12.43 (18–79)^a^	0.949
Sex			0.160
Female	13 (59.1%)	14 (40.0%)	
Male	9 (40.9%)	21 (60.0%)	
Type of lesion			0.000*
Plague	3 (13.6%)	0 (0.0%)	
Red Nodule	14 (63.6%)	2 (5.7%)	
Other palpable	3 (13.6%)	29 (82.9%)	
Nodule			
Multinodular	2 (9.1%)	4 (11.4%)	
Anatomic location			0.725
Head and neck	2 (9.1%)	4 (11.4%)	
Trunk	18 (81.8%)	30 (85.7%)	
Extremities	2 (9.1%)	1 (2.9%)	
Immunohistochemical			
CD34(+)	23	35	

*NS* no significance, *DSFP* Dermatofibrosarcoma Protuberans

*, *P*<0.05

^a^ Mean ± SD (range)

The mean age at diagnosis was similar between the primary DFSP group (42.3 ± 10.9 years) and the recurrent DFSP group (38.1 ± 12.4 years) and ranged from 18 to 79 years. There were no statistically significant differences in sex. The incidence was not different between males and females in either group. There were statistically significant differences in the type of lesion (*p* < 0.050). Red nodules on the skin (63.6%; 14 of 23) were found more frequently in the primary DFSP group, while other palpable nodules (82.9%; 29 of 35) were found more frequently in the recurrence DFSP group.

The most common site was the trunk (81.8% in the primary DFSP group; 85.7% in the recurrence DFSP group), followed by the head and neck (9.1% in the primary DFSP group; 11.4% in the recurrence DFSP group), and the extremities (9.1% in the primary DFSP group; 2.9% in the recurrence DFSP group). All patients in both groups were positive for CD34.

### Imaging findings

**Fig. 1:**
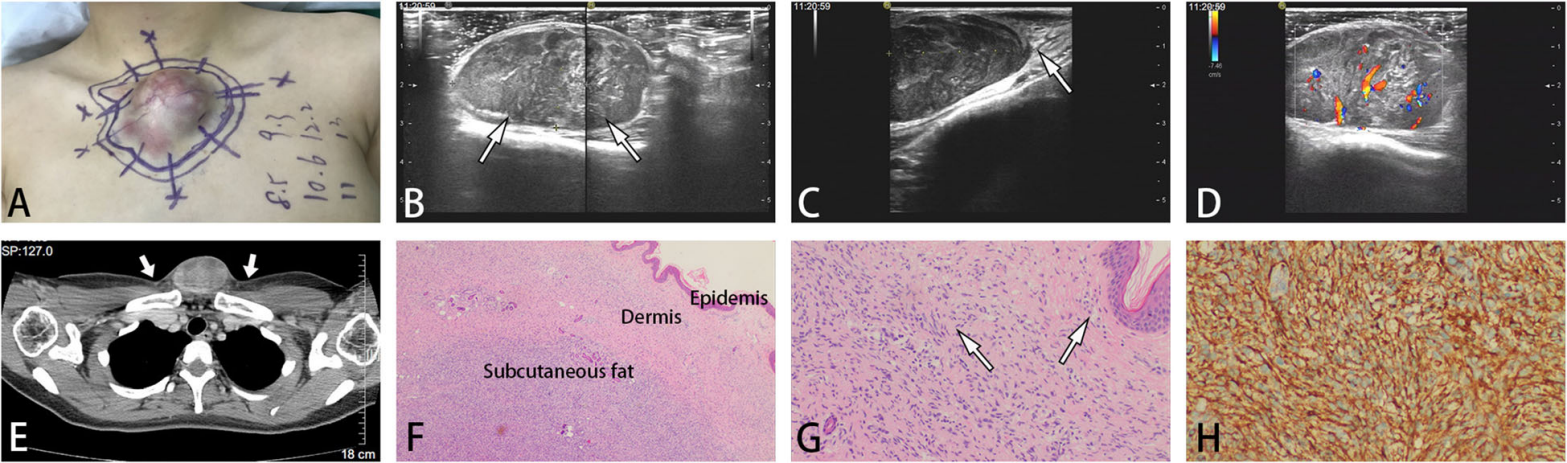
**A**, A purplish red plaque protruding above the skin for more than 20 years showed rapid enlargement in the last 9 months. **B**, US showed an ovoid circumscribed subcutaneous lesion with mixed internal hypoechogenicity. **C**, Superficial tapering at the lesion/skin interface was observed, forming tentacle-like projections or a “claw” sign (arrow), and **D**, color Doppler sonography demonstrated a marked intralesional color signal. **E**, CT images showed nonhomogeneous enhancement and tentacle-like projections or a “claw” sign (arrow). **F**, **G**, Hematoxylin and eosin staining revealed that the primary DFSP lesions involved the overlying dermis and hypodermis (arrow) (F; H&E, 25×, G; H&E, 100×). **H**, Immunohistochemical staining was positive for CD-34 (original magnification 200×)

**Fig. 2:**
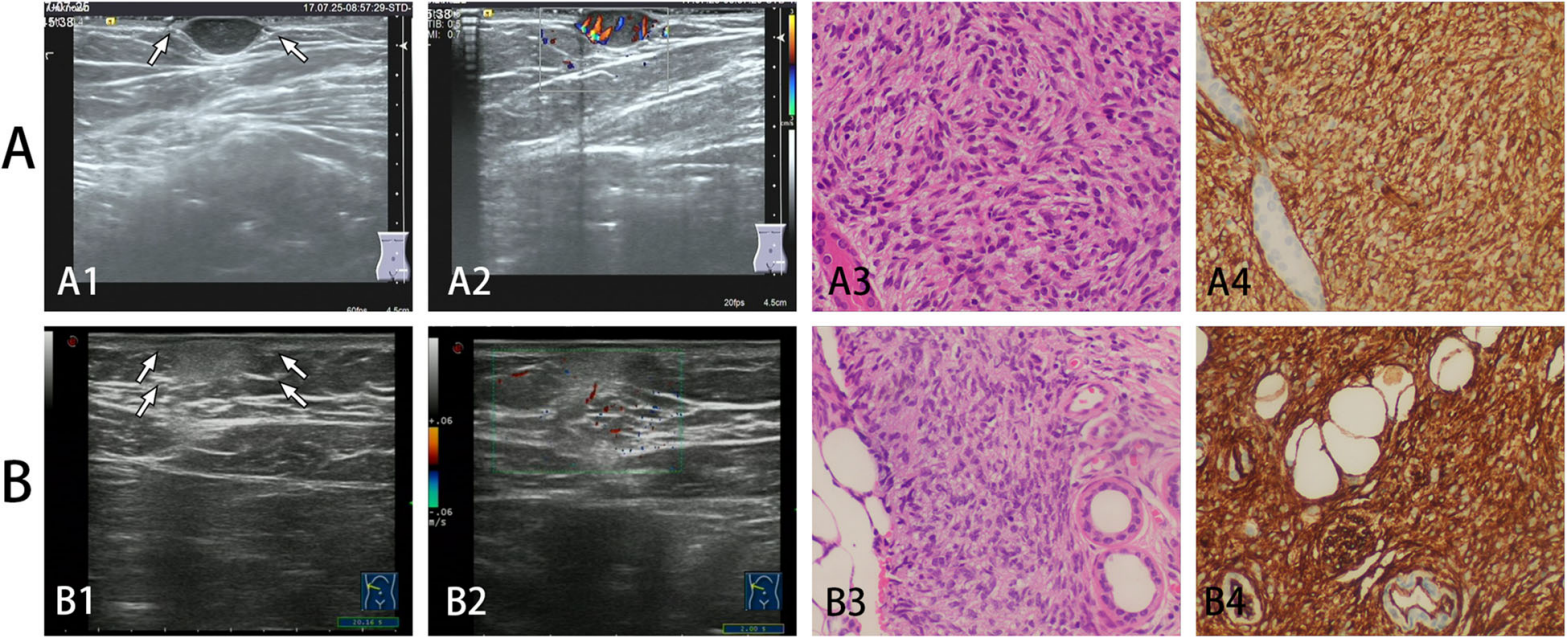
**A** A 25-year-old man with primary DFSP in the back. **B** A 32-year-old female with primary DFSP in the abdominal wall. **A1, A2** US showed an ovoid circumscribed subcutaneous lesion with profuse blood flow throughout and hypoechogenicity, forming tentacle-like projections or a “claw” sign (arrow). **A3** Hematoxylin-eosin staining showing high cellularity with slender spindle cells arranged in a distinct storiform pattern (H&E, 100×). **A4** Immunohistochemical staining was positive for CD-34 (original magnification 200×). **B1, B2** US showed a poorly defined hyperechoic mass with restricted blood flow in the subcutaneous soft tissue. The margin of the tumor appeared to have pseudopodial-like protrusions, forming tentacle-like projections or a “claw” sign (arrow). **B3** Hematoxylin-eosin staining showing that tumor cells infiltrated into the surrounding subcutaneous fat tissue H&E, 100×). **B4** Immunohistochemical staining was positive for CD-34 (original magnification 200×)

**Fig. 3:**
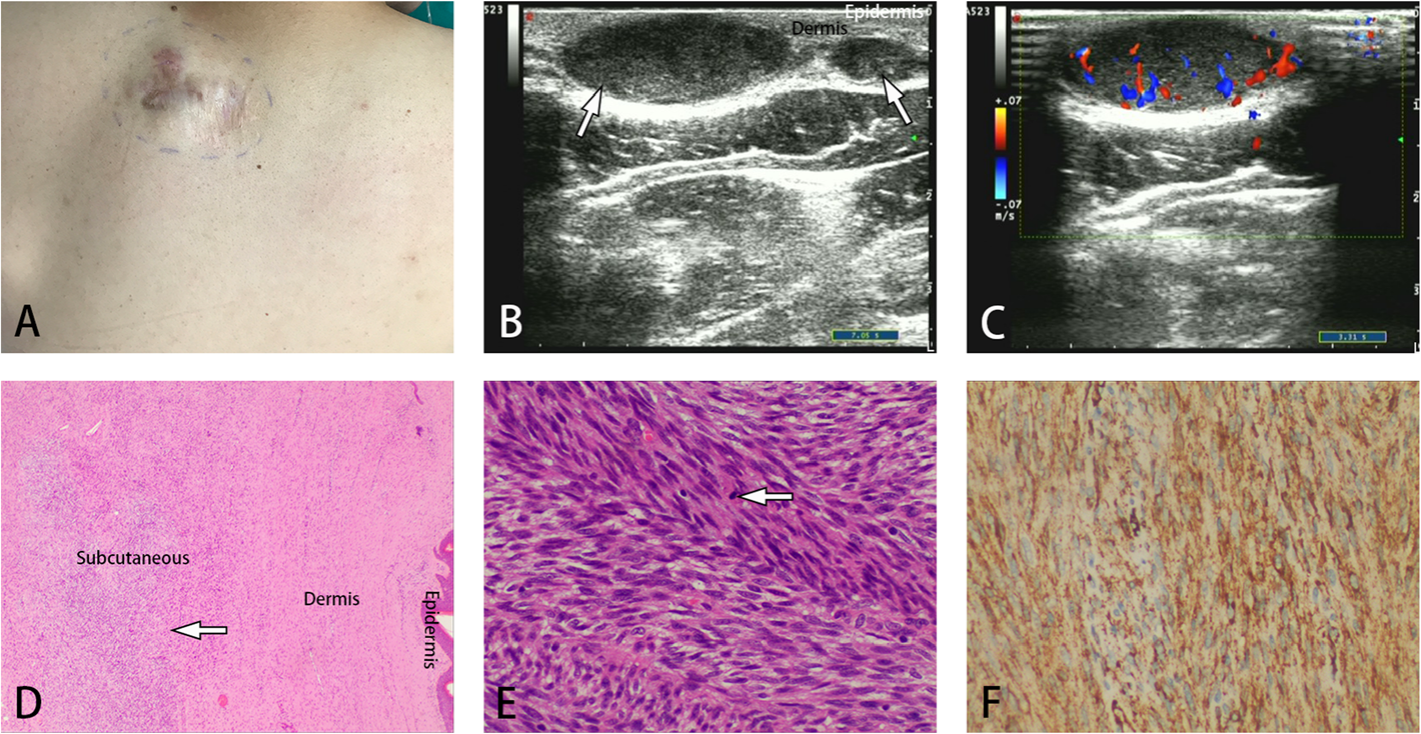
A 25-year-old man with recurrent DFSP in the back. **A**, Erythematous nodules at and near the old scar on the back were observed 6 years after surgery. **B**, US showed multiple ovoid lesions with hypoechogenicity forming tentacle-like projections or a “claw” sign (arrow). **C**, Color Doppler sonogram showing profuse blood flow throughout the mass. **D**, **E**, Hematoxylin-eosin staining showing high cellularity with slender spindle cells arranged in a distinct storiform pattern accumulating mainly in the underlying soft tissue

The imaging features are summarized in Table 2. For the cases studied, the primary DFSP tumor sizes at presentation ranged from 5.0 mm to 80 mm, while the recurrent DFSP tumor sizes ranged from 6.5 mm to 50 mm. Most lesions in both groups were single lesions (primary DFSP group: 90.9%; recurrence DFSP group: 88.6%) and involved the skin and underlying soft tissue at the same time. Most of the primary DFSP lesions involved the overlying dermis and hypodermis (86.4%; 19 of 23) but not deeper subcutaneous structures (Fig. 1). However, fixation to more deeply seated structures was often observed at the original surgical incision in recurrent cases of DFSP (62.9%; 22 of 35) (Fig. 2). DFSP generally exhibits the following US morphologies: regularly oval, tentacle-like projections or a “claw” sign, lobulated, and an irregular shape. There were statistically significant differences in US tumor morphology between the two groups (*p* < 0.050). The lesions in the primary DFSP group (50.0%; 11 of 23) showed tentacle-like projections or a “claw” sign, while the lesions in the recurrent DFSP group were regularly oval (28.4%; 11 of 35), lobulated (22.9%; 8 of 35) and irregular in shape (34.3%; 12 of 35) (Figs. 1, 2, and 3).

**Table 2:** Characteristics on US of DSFP

Characteristic	Primary DSFP (*n* = 23)	Recurrent DSFP (*n* = 35)	*P*-value
Tumor size (mm)	21.84 ± 12.20 (6.5–50.33)^a^	25.10 ± 20.31 (5.0–80.33)^a^	0.093
Number of lesion			1.000
Single	20 (90.9%)	31 (88.6%)	
Multiple	2 (9.1%)	4 (11.4%)	
Lesion location			0.000*
D-H	19 (86.4%)	13 (37.1%)	
Hypodermis	3 (13.6%)	22 (62.9%)	
US morphology			0.038*
Regularly oval	3 (13.6%)	10 (28.4%)	
Tentacle-like projections or “claw” sign	11 (50.0%)	5 (14.3%)	
Lobulated	4 (18.2%)	8 (22.9%)	
Other Irregular Shape	4 (18.2%)	12 (34.3%)	
US echogenicity			0.455
Hypoechoic mass	12 (54.6%)	25 (71.4%)	
Mixed hypo	7 (31.8%)	7 (20.0%)	
Hyperchoic	3 (13.6%)	3 (8.6%)	
Blood flow on CDFI			0.28
Rich (Alder0-1)	5 (22.7%)	19 (54.3%)	
Not rich (Alder2-3)	17 (77.3%)	16 (45.7%)	
Metastases	0	1	NS
US diagnosis			NS
Benign lesion	8 (34.8%)	0 (0.0%)	
Malignant/recurrence lesions	4 (17.4)	31 (88.6)	
Indeterminacy	11 (47.8%)	4 (11.4%)	

*D-H* dermis and hypodermis, *US* ultrasound, *CDFI* color Doppler flow, *hyper* hyperechoic, *hypo* hypoechoic, *Mixed hypo* a mass with mixed hypoechoic and hyperechoic regions, *NS* no significance

*, *P*<0.05

^a^ Mean ± SD (range)

There was no statistically significant difference in tumor echo between the two groups. DFSP lesions appeared as a hypoechoic mass (54.6% in the primary DFSP group; 71.4% in the recurrence DFSP group) and a mass with mixed hypoechoic or hyperechoic regions. In addition, the primary DFSP lesions showed more mixed hypoechogenicity than the recurrent DFSP lesions. In our study, hypervascularity (blood flow on CDFI) was common in both groups (Figs. 1, 2, and 3). The diagnostic accuracy in the recurrence DFSP group was significantly higher than in the primary DFSP group(88.6% in the recurrence DFSP group; 17.4% in the primary DFSP group).

**Fig. 4:**
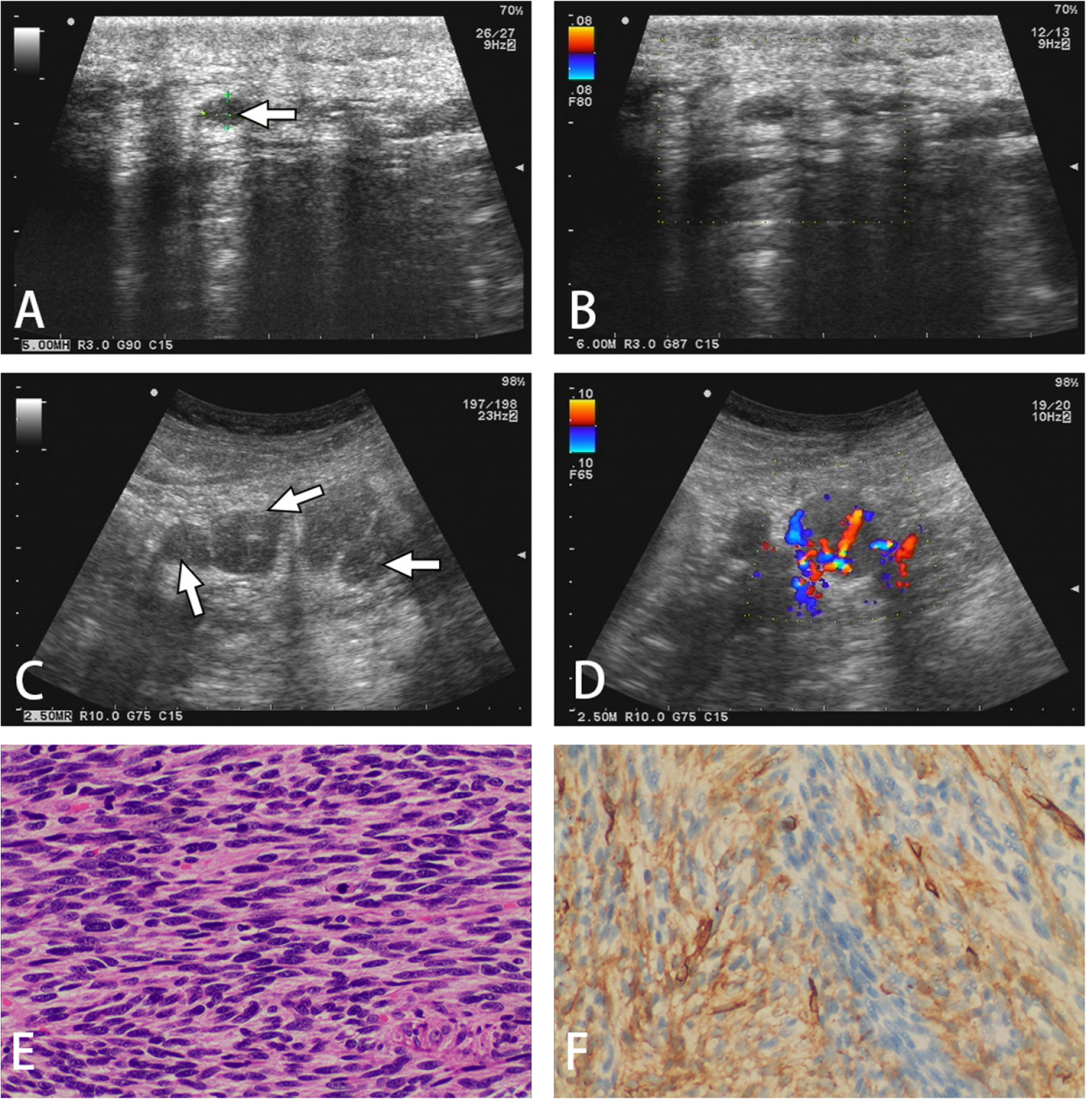
A 61-year-old man with recurrent and metastatic DFSP. **A, B,** An ovoid nodule with poor blood flow at and near the old scar on the back was observed 7 years after surgery. **C, D,** Multiple retroperitoneal metastases of DFSP with rich blood flow were detected over three years of follow-up after a second wide local excision. **E**, H&E staining (200×) showing spindle cells in the retroperitoneal mass arranged in a storiform pattern. **F**, Immunohistochemical staining was positive for CD-34 (original magnification 200×)

Among these patients, a 61-year-old male with recurrent DFSP on the back with multiple retroperitoneal metastases was observed. The patient with distant metastasis was followed up 3 years after a second wide local excision (shown in Fig. 4).

## Discussion

DFSP is a rare soft-tissue malignancy that accounts for less than 0.1% of all malignancies and less than 1% of all soft-tissue sarcomas and is locally aggressive and slow growing [13, 14]. It has very low metastatic potential and a high risk of local recurrence if not treated properly, in agreement with our results. The exact etiology of DFSP is not fully understood. Some studies have reported that genetic factors play an important role in the pathogenesis of DFSP, which is characterized by the chromosomal translocation t(17;22)(q22;q13) [15]. According to the majority of studies, the most common location of DFSP is the trunk [16]. The lower and upper extremities followed by the head and neck region are the next most common sites of DFSP. It most frequently occurs in young and middle-aged patients between 25 and 45 years of age, with a mean age between 40 and 43 years [17]. Our results were in accordance with those of previous studies. For primary DFSP, a slow-growing, small, firm, painless, skin-colored dermal plaque and protuberant lesion are typically observed in the early stages. For recurrent DFSP, palpable subcutaneous nodules or subcutaneous masses at the original surgical incision are observed. According to most reports, the incidence of DFSP is higher in women than in men, but these results have been inconsistent in some studies [18]. In our study, there was an equal distribution between males and females.

The diagnosis of DFSP is usually based on clinical appearance only; however, in most cases, the clinical and pathological manifestations of DFSP are atypical. Therefore, DSFP is a troublesome disease for many doctors. Therefore, imaging examination can be used as an auxiliary examination means for the diagnosis of DFSP and can also improve the accuracy of the diagnosis and provide evidence for further treatment. MRI, CT, and US are the most commonly utilized imaging methods for DFSP diagnosis and assessment [3, 8, 11]. MRI and CT are useful for assessing the extent of involvement, particularly for recurrent disease or large and atypical primary lesions [19]. Preoperative MRI may help with surgical planning. Additionally, chest CT should be performed to screen for pulmonary metastases in more advanced or recurrent cases [3]. DFSP is frequently small and superficial because it progresses slowly over a long period of time before entering a rapid growth phase. US is quick, inexpensive, non-invasive, and accessible [20]. Additionally, Doppler techniques can increase the specificity of US by providing a real-time evaluation of vascularity [7]. Therefore, it is commonly utilized for the initial assessment of superficial soft-tissue masses, including DFSP, and can be used as a routine examination for DFSP.

DFSP appears to be derived from dermal fibroblasts. It is characterized by a uniform population of spindle-shaped fibroblasts arranged in a storiform “cartwheel” pattern along with a fibrous stroma background under a microscope [18, 21]. Some documents have pointed out that the characteristic imaging findings of DFSP obtained by US are closely related to its pathologic findings, such as marginal infiltration and tumor composition [22]. Shin YR et al. [8] reported that pathologically, samples corresponding to hypoechoic DFSP were composed primarily of tumor cells, and samples corresponding to mixed echogenic DFSP were composed of tumor cells and fibrous tissues, which is in agreement with our results. Rodriguez BA et al. retrospectively analyzed the data of 3 new cases and reviewed the literature of DFSP and found a “jellyfish-like” sonographic pattern (an oval-shaped dermal body parallel to the epidermis and focal ill-defined limits invading the subcutaneous tissue through interconnected tentacle-like projections) that can be used as a useful adjunct in formulating the diagnosis of DFSP [10]. However, in our cases, the “jellyfish-like” sonographic pattern was not very common. We observed that the anterior and posterior boundaries of most masses were relatively smooth, while the peripheral boundary was not clear (Figs. 2, 3). This is in agreement with the findings of Sung et al. [23]. They observed a characteristic radiological “claw” sign featuring the interface between the lesion and the skin in various imaging modalities, including US, CT, and MRI. The “claw” sonographic pattern was more common in the primary DFSP group than in the recurrent DFSP group. Other US morphologies of DFSP are a lobulated morphology with an oval body and one or more peripheral lobulations and a regular oval morphology with no lobulations or projection. These morphologies may represent a different stage in evolution or simply an altogether different growth pattern. Is this phenomenon related to local recurrence? We expect that with increasing acceptance and wider use of new techniques, drawing more definitive conclusions will be possible in the near future.

Surgical excision of the lesion is the standard treatment for DFSP. Some reports [24–26] have noted that the recurrence rate after local excision was significantly higher than that after wide-margin excision (margins over 3 cm), while the recurrence rate after wide-margin excision was significantly higher than that after Mohs surgery. Preoperative US imaging may assist in surgical planning and help to prevent local recurrence. Although US imaging findings for DFSP are nonspecific, they may help to define the boundaries. The use of preoperative enhanced US to improve the accuracy of surgical resection of DFSP was reported by Chuan Ma [24–26].

DFSP is nonspecific and can easily be confused with other superficial masses such as epidermal cysts, pilomatricoma, lipoma, and dermatofibroma [11, 20]. The differential diagnosis of DFSP from other superficial soft-tissue tumors is very important because the treatment strategies and prognoses differ [8, 11]. Epidermal cysts are debris-filled sacs that generally appear as well-defined oval shapes with internal floating echoes on the US. The US is very helpful in differentiating between cystic and solid lesions. DFSP is hypoechoic or mixed echogenic and not floating. Furthermore, on CDFI, epidermal cysts usually have no blood flow, while DFSP tumors often have plenty of color signals within and around the lesion, as described in Table 2 [8, 20]. Pilomatricoma, originating from hair cortex cells, shows a well-defined mass with inner echogenic foci and a peripheral hypoechoic rim or a completely echogenic mass with strong posterior acoustic shadowing because of calcification in the subcutaneous layer. However, no calcification was observed in any of the DFSP patients, and most lesions had posterior reinforcement [8, 20]. Lipoma often occurs in the subcutaneous adipose tissue of the limbs and is derived from mature adipose tissue [8, 20]. Lipoma typically appears as a well-defined oval or pad-like mass with fine linear striations and little or no increased blood flow. Dermatofibroma, whether single or multiple, occurs in various parts of the limbs, accompanied by thickening of the skin tissue. On the US, dermatofibromas are usually visible as avascular dermal lesions and are characterized by ill-defined margins, marginal spiculation, and changes in the echogenicity of the surrounding soft tissues [20, 27].

There are a few limitations to our study. First, the number of patients in the study was small due to the rarity of DFSP. Second, because the inclusion of DFSP patients and examinations were performed at different times, the US imaging methods were not standardized, which is a limitation of the retrospective nature of the study.

Another limitation was that we did not use a gel-pad to optimize the US resolution. Color Doppler technology plays a very important role in blood flow detection of superficial tumors. Corvino A et al. [7] found that the use of a gel stand-of pad allows the detection of otherwise missed peri- or intra-lesional flow signals on Doppler imaging, increasing the diagnostic role of this technique in the differential diagnosis of superficial lesions. Third, we compared only the US imaging features of DFSP, and we did not integrate the basal US examination with contrast-enhanced ultrasound (CEUS). CEUS consists of an injection of gas-filled microbubbles and is able to depict in real time the microvascularity and neoangiogenesis of the lesion, increasing confidence in diagnosing a tumor. In future work, we will continue to collect more cases of DFSP by appropriate use of the gel stand-of pad and focus on imaging features via CEUS, CT, and MRI.

## Conclusions

In summary, US appears to be a useful, noninvasive tool in the diagnosis of DFSP. Primary DFSP can involve the overlying dermis and hypodermis, more frequently appearing as a hypoechoic mass with tentacle-like projections or a “claw” sign. For recurrent DFSP, palpable subcutaneous nodules or subcutaneous masses at the original surgical incision and regularly oval, lobulated, and irregular nodules are more commonly observed. All lesions in both groups exhibited hypervascularity. In addition, we must pay attention to clinical information such as the age of onset, disease course, lesion site, and the appearance of skin nodules. If doctors can accurately diagnose this disease, DFSP patients can avoid suffering from additional pain. Therefore, further study is necessary to evaluate the role that the US plays in the diagnosis of DFSP.

## Data Availability

The datasets used and/or analyzed during the current study are available from the corresponding author on reasonable request.
